# Supplementary material for: Pre- and post-experimental manipulation assessments confirm the increase in number of birds due to the addition of nest boxes
Source: PeerJ. 2016 Mar 15;4:e1806. doi: 10.7717/peerj.1806 (PMC4797770; doi:10.7717/peerj.1806)
Supplement: Supplemental Information 1 — List of geographical coordinates of the sampling points. [file peerj-04-1806-s004.doc]

**Location of the experimental plots**

**Javier Mina (1)**

19º 11’ 29.26’’N

97º 57’ 45.06’’ O

19º 11’ 17.92’’N

97º 57’ 52.12’’ O

19º 11’ 39.60’’N

97º 58’ 2.91’’ O

19º 11’ 27.76’’N

97º 58’ 9.45’’ O

**Ixtenco (2)**

19º 14’ 33.28’’N

97º 59’ 8.20’’ O

19º 14’ 20.47’’N

97º 59’ 8.04’’ O

19º 14’ 20.57’’N

97º 59’ 25.81’’ O

19º 14’ 33.56’’N

97º 59’ 26.38’’ O

**Albergue (3)**

19º 16’ 28.09’’N

98º 2’ 8.45’’ O

19º 16’ 40.28’’N

98º 2’ 11.98’’ O

19º 16’ 35.58’’N

98º 2’ 25.87’’ O

19º 16’ 23.48’’N

98º 2’ 22.18’’ O

**Pilares (4)**

19º 15’ 38.92’’N

97º 58’ 2.91’’ O

19º 15’ 26.42’’N

97º 58’ 2.94’’ O

19º 15’ 38.67’’N

97º 58’ 20.57’’ O

19º 15’ 26.25’’N

97º 58’ 20.54’’ O

**Altamira (5)**

19º 16’ 54.44’’N

98º 0’ 3.20’’ O

19º 16’ 41.83’’N

98º 0’ 3.00’’ O

19º 16’ 44.66’’N

98º 0’ 18.04’’ O

19º 16’ 57.04’’N

98º 0’ 18.13’’ O

**Teacalco (6)**

19º 17’ 24.33’’N

98º 2’ 22.05’’ O

19º 17’ 39.73’’N

98º 2’ 29.05’’ O

19º 17’ 33.93’’N

98º 2’ 39.32’’ O

19º 17’ 18.63’’N

98º 2’ 32.49’’ O
